# The ethics of inattention: revitalising civil inattention as a privacy-protecting mechanism in public spaces

**DOI:** 10.1007/s10676-020-09575-7

**Published:** 2021-01-16

**Authors:** Tamar Sharon, Bert-Jaap Koops

**Affiliations:** 1 Faculty of Philosophy, Theology and Religious Studies, Radboud University, Nijmegen, The Netherlands; 2 Tilburg Institute for Law, Technology, and Society (TILT), Tilburg University, Tilburg, The Netherlands

**Keywords:** Civil inattention, Privacy, Facial recognition, Social norms, Discretion, Reserve

## Abstract

Societies evolve practices that reflect social norms of appropriateness in social interaction, for example when and to what extent one should respect the boundaries of another person’s private sphere. One such practice is what the sociologist Erving Goffman called civil inattention—the social norm of showing a proper amount of indifference to others—which functions as an almost unnoticed yet highly potent privacy-preserving mechanism. These practices can be disrupted by technologies that afford new forms of intrusions. In this paper, we show how new networked technologies, such as facial recognition (FR), challenge our ability to practice civil inattention. We argue for the need to revitalise, in academic and policy debates, the role of civil inattention and related practices in regulating behaviour in public space. Our analysis highlights the relational nature of privacy and the importance of social norms in accomplishing and preserving it. While our analysis goes some way in supporting current calls to ban FR technology, we also suggest that, pending a ban and in light of the power of norms to limit what is otherwise technically possible, cultivating new practices of civil inattention may help address the challenges raised by FR and other forms of digital surveillance in public.

## Introduction

You hop on a crowded tram and quickly scan your surroundings; no one you know, nothing out of the ordinary. You grasp a pole, thereby securing your stance and your claim on a temporary, yet evident personal space, while trying to position yourself so as not to impose yourself on the personal space of others. The number of passengers makes this difficult, so you do your best to show that you are not unduly interested in anyone around you: ostentatiously turning your head away from the self-help magazine the middle-aged woman nearby is reading; not allowing your eyes to stop on the provocative text on the teenagers’ T-shirt a few poles down; focusing on the list of tram stops on the wall instead of the marital argument your neighbour is conducting on his phone. Other passengers know the drill and do the same, taking in your presence as an additional component of the public space they now share with you, before turning their attention back to the city outside the window, their book or their smartphone. Those engrossed by their smartphones usually peer down into them, reading, texting, playing. Others hold them up in front of them, conversing with someone in a distant place, checking their make-up, or taking a photo. Actually—while she didn’t seem to be looking at you at all, could it be that that woman a few seats down just took your photo? And is she using it to identify you against a database of faces?

As this scenario depicts, the social norm of showing a proper amount of indifference to others—what the sociologist Erving Goffman (1963) called “civil inattention”—is a commonplace but potent privacy-preserving mechanism, which people routinely practice in everyday situations of physical proximity. It contributes to accomplishing the value of privacy in public. Indeed, values, as pragmatist ethicists underscore, acquire concrete meaning in acts; in routine practices and social norms which are recognizable to others (Dewey 1954). Thus, keeping a promise enacts the value of trust, waiting in line enacts the value of fairness, and demonstrating civil inattention enacts the value of privacy. Here, morality exists first and foremost in the form of actions, and typically actions that seem so self-evident that we barely pay attention to them (Gouinlock 1994). But new technologies frequently disrupt routine moral practices and taken-for-granted norms, hindering the possibility to enact values in customary ways (Keulartz et al. 2004; Sharon 2017; Swierstra 2013). When this happens, new technologies solicit a variety of responses in an attempt to restabilise a moral landscape (Swierstra 2013)—limiting what a technology can do (design), codifying what uses a technology can be put to (regulation), or cultivating new ways of practicing a norm (behaviour).

In this paper we show how new networked technologies, such as the facial recognition (FR) technology alluded to in the scenario above, add new dimensions to our social interactions that impair customary practices of civil inattention and associated privacy-protecting norms of discretion, nonacknowledgment, reserve and disattendability. We argue that a focus on civil inattention, a heretofore undervalued and underdeveloped concept in the academic and policy debate on privacy and surveillance technologies, can both extend our understanding of what kind of privacy problems surveillance technologies raise and offer additional means for addressing these. Namely, our analysis highlights the relational nature of the value of privacy and the importance of social norms in accomplishing and preserving it (Kudina and Bas 2018; Roessler and Mokrosinska 2013; Tonkiss 2003).

The paper is structured as follows. We begin with an analysis of the concept of civil inattention and correlated norms, and their function as privacy-preserving mechanisms. Next, we identify the challenge posed by consumer-based use of FR as a behavioural-informational privacy problem (Koops et al. 2017) and describe how the affordances of this technology impair the practice of civil inattention, which seeks precisely to preserve behavioural-informational privacy. We then discuss the benefits and shortcomings of dominant theoretical approaches and legal instruments in the privacy debate for addressing the privacy problem raised by consumer-based FR. We suggest that a civil inattention approach to privacy better accounts for the collaborative, relational nature of privacy in public and entails a more realistic distribution of responsibilities with regard to its achievement. We conclude with a discussion on the need to revive the concept of civil inattention in both scholarly and regulatory debates on surveillance technologies, and some of the limitations of our own analysis. Finally, we suggest that—pending a complete ban on the use of FR in public spaces, and in light of the power of norms to limit what is otherwise technically possible—cultivating new practices of civil inattention in digitally pervasive environments may help address some of the challenges raised by ubiquitous digital surveillance.

## Civil inattention as a privacy-preserving mechanism

Erving Goffman (1963) coined the term civil inattention to denote the practice of acknowledging strangers with whom we come in close proximity, all the while displaying disinterestedness.^1^ When two people are mutually present but not involved in focused interaction, such as strangers passing each other by on the street, Goffman explains that three types of behavioural patterns are at their disposal. They can stare openly and fixedly at one another, they can ignore one another, and they can glance at each other and quickly avert their gaze. The first two types of behaviour, explains Goffman, are inappropriate, insofar as they constitute others as “non-persons”—either as objects of scrutiny or revulsion (a category that is more befitting to animals or freaks), or as objects not worthy of any interest at all. Civil inattention, the third option, is the one we deem “proper” according to Goffman. It involves suspending specific attention to others and their behaviours and characteristics. It implies treating the stranger as a person, by acknowledging their existence without lingering on it.

For Goffman, within this small and subtle act lies a world of civility and respect that addresses the challenge of making life in complex modern societies bearable. He writes: “We have here what is perhaps the slightest of interpersonal rituals, yet one that constantly regulates the social intercourse of persons in our society” (Goffman 1963, p. 84). Extending civil inattention to others is a means of communicating that one is neither hostile towards them, exemplified for Goffman in the Southern “hate stares” of whites to blacks, nor that one wants to avoid them, as one might avoid beggars or psychiatric patients on the streets. It shows that one is neither offensive nor defensive, but impartial: a display of disinterestedness without disregard, acknowledgment without recognition, availability without imposition, of contact without intimacy or hostility. In modern Western societies,^2^ civil inattention is a norm that fosters privacy within public spaces. It helps regulate the increased accessibility and availability of persons in situations of co-presence, by erecting boundaries where they do not physically exist.

### The delicate choreography of civil inattention

Civil inattention is a very intricate mechanism. It does not only involve the double requirement of displaying disinterestedness without disregard on the part of the individual who *offers* civil inattention; it is also part of a larger choreography of interpersonal relations that includes a requirement on the part of that individual who *enjoys* civil inattention. If there is a certain obligation to extend civil inattention to others, and thus a certain “right” to civil inattention, for Goffman this is not a natural right but one that is acquired, by behaving in a manner that summons or deserves civil inattention as an adequate response. “To behave properly and to have the *right* to civil inattention are related”, writes Goffman: “propriety on the individual’s part tends to ensure his being accorded civil inattention” (1963, p. 87). Indeed, while it may be possible to curtail one’s sight, other senses are more difficult to rein in; ears cannot always help but hear, and noses certainly cannot help but smell. The counter-part of civil inattention is thus always some form of disattendability—the status of not giving cause for a particular need for attention. For Goffman, one needs to earn civil inattention, by making oneself *civilly disattendable*, by not forcing oneself on the attention of others through extreme impropriety. This is achieved in behaviour (e.g., not seeking the gaze of others, speaking softly), and can be aided by material props, or what Goffman calls “involvement shields”: fans, newspapers, books, etc., which act as barriers to perception. Note that Goffman links disattendability to avoiding *non-extreme* impropriety: it is precisely with non-extreme forms of impropriety (such as picking one’s nose, sneezing, walking around with a tear in one’s trouser bottoms) that civil inattention works to smooth relations in public, and to enable a basic level of privacy.

**Fig. 1:**
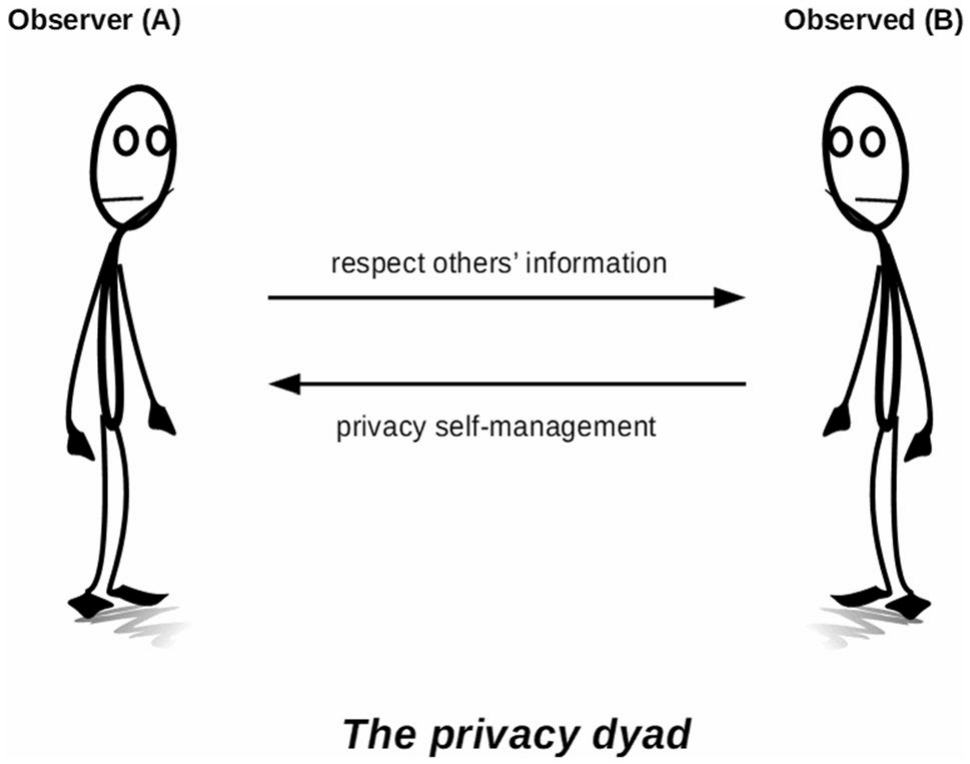
The privacy dyad

For Goffman, then, as for other privacy scholars writing today on the relational and social nature of privacy (see e.g. Roessler and Mokrosinska 2013), privacy is something that needs to be accomplished within a relationship. It can be helpful to visualise this in terms of a simplified illustration of the relationship between two strangers in public—a privacy dyad—where person *A* is in a position to observe person *B*, and where person *A* should respect person *B’*s privacy, at the same time that person *B* should engage in some privacy self-management (Fig. 1). In Goffman’s terminology, person *A* should extend or grant civil inattention to person *B*, while person *B* should at the same time engage in civil disattendability.

### Correlated norms

Goffman was not the first nor the only social theorist interested in identifying the system of implicit rules and behaviours that regulate social order in situations where strangers are accessible to one another (see also Arendt 1958; Benjamin 2002; Simmel 1906, 2002; Wirth 1938). And a number of correlated norms including “indifference”, “reserve”, “discretion”, “nonacknowledgment”, “avoidance”, “social distance” and “reticence”, have been articulated by others. Here we briefly discuss several of these concepts in order to emphasise the role of social norms in privacy protection and to better understand the role of civil inattention among and in relation to these other concepts and mechanisms.

Georg Simmel, for example, preoccupied by the state of individuality in metropolises at the beginning of the twentieth century, analysed ways in which city dwellers develop psychological coping mechanisms to protect themselves from the sensory overload of big cities and to enforce a distance between themselves and others. In “The Metropolis and Mental Life” (2002), he addresses what he maintains is one of the deepest problems of modern life, the need for the individual to protect her inner life from being “swallowed up” by the city. The intensification of sensual stimuli in the city as compared to rural settings creates a situation in which the individual must develop ways of buffering herself from her surroundings. Closer to civil inattention, at the level of the individual and her environment, what Simmel calls a “blasé outlook” is one such barrier, an attitude of indifference and de-sensitisation towards the constant bombardment of stimuli of the city. At the level of interpersonal relations, individuals practice what Simmel calls “reserve”, a mental attitude that creates distance from the unceasing impressions emanating from other persons. Reserve ensures a degree of personal freedom to the individual from the oppressive closeness characteristic of smaller communities. In this case it involves not taking in too much, in situations where other individuals (and the city), fail to practice what Goffman would call sufficient “disattendability”. Differently from civil inattention, however, Simmel’s reserve is about the retention of one’s own privacy vis-à-vis others. In terms of our privacy dyad, it aims to protect *A* from an absence of disattendability on the part of *B* (and on the part of the city in general). Yet in Simmel’s writings too, several interacting norms are required for making public life in cities bearable.

Reserve is also about not seeking out more information than is willingly disclosed. Simmel discusses this form of “general reserve” or “discretion” in “The Sociology of Secrets and Secret Societies” (1906) in relation to secrecy, which he maintains is a necessary component of intimate personal relations and of social life in modern society. Discretion involves restraining oneself from knowing more about others than they expressly wish to reveal. For Simmel, an ideal sphere of discretion surrounds every human being, which cannot be penetrated without challenging the value of the other person. To penetrate this sphere constitutes “a violation of the personality … a violation of the ego at its centre” (1906, p. 454). Discretion is thus closer to Goffman’s civil inattention than the blasé attitude or reserve, insofar as it is first and foremost a norm that *A* applies to enable privacy protection for *B*, rather than one that *A* erects in order to distance herself from *B*.

Alan Westin (1967, p. 32), the renowned privacy scholar, draws on Simmel’s “reciprocal reserve and indifference” to articulate what he designates as the “fourth and most subtle” state of privacy—“reserve”.^3^ He defines this as “the creation of a psychological barrier against unwanted intrusion; this occurs when the individual’s need to limit communication about him is protected by the willing discretion of those surrounding him” (1967, p. 32). Thus, Westin seems to collapse the dual mechanism of reserve and discretion into a single social practice, through which people can achieve the privacy state of what he calls “reserve”. The importance of this social behaviour for privacy in public is highlighted where Westin states that the “manner in which individuals claim reserve and the extent to which it is respected or disregarded by others is at the heart of securing meaningful privacy in the crowded, organization-dominated settings of modern industrial society and urban life” (1967, p. 32).

In the 1990s, prompted by the press’s handling of the Clinton–Lewinsky affair, the philosopher Thomas Nagel offered an analysis of the social and psychological function of privacy norms in his essay “Concealment and Exposure” (1998). Nagel identifies “reticence”, similar to Goffman’s disattendability and Simmel’s reserve, and “nonacknowledgment”, similar to Goffman’s civil inattention and Simmel’s discretion, as the two correlate norms that govern privacy-respecting relations in public. Nagel’s starting point, like Simmel’s, is that both individual sanity and civilization are at stake in the chaotic profusion of impressions emanating from persons, the inner lives of which are so confused and rich that uncensored exposure would make collective life impossible. For Nagel, it is the convention of reticence, the screening out of our thoughts, desires and impulses, that is in particular in need of revival in our culture of forced exposure, more than nonacknowledgment. We need to re-learn how to curtail our unruly, private selves so as not to impose them on others in public. According to Nagel, reticence erects a boundary between public and private matters, which then allows for nonacknowledgement: appropriate collective responses to “what remains individual and may be ignored” (1998, p. 7) to take place.

**Fig. 2:**
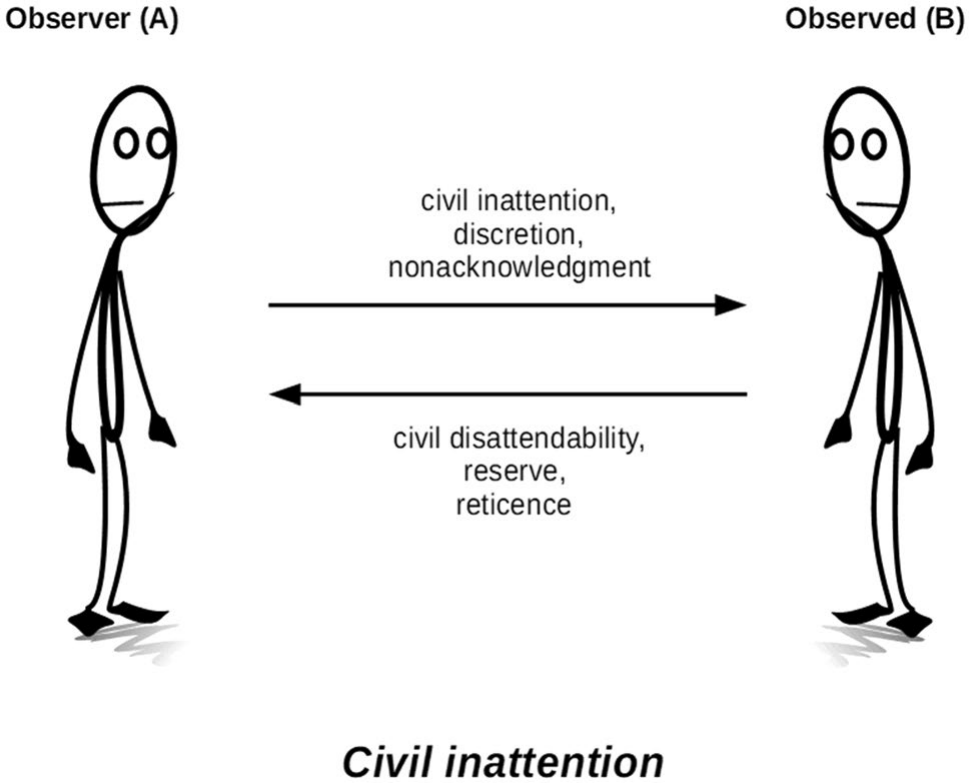
Civil inattention

Like Goffman, these authors all theorize privacy-protecting social practices as a dual, reciprocal and collaborative effort that involves some form of restraint on both what is exposed and what is sought out: where *B* demonstrates reserve, reticence, or disattendability, she can reasonably expect *A* to exercise discretion, nonacknowledgment, or civil inattention (Fig. 2). For all of these authors, privacy emerges as an important component of social relationships and social order that requires quite some work to be accomplished.

Although these mechanisms are largely similar, there are subtle but meaningful differences between the various concepts. Discretion is associated with knowledge (indicating restraint to gather information about another’s private life), whereas civil inattention is associated primarily with visibility or other forms of sensorial perceptibility (indicating restraint to register the impressions that another gives off). Nonacknowledgment indicates a general state of indifference, which can be good or bad, or more or less justified, whereas civil inattention suggests achieving the socially desirable level of indifference that the situation requires.

Another, and more fundamental, difference is that Goffman’s concept of civil inattention does not only denote the act of exercising restraint towards observing another, but also the act of *showing* this exercise of restraint towards observing another. The show, in much of Goffman’s work, is a key part of the performance of social behaviour. It is the fleeting eye contact, or the slight raising of the head when stepping aside on the kerbside to let someone pass, that acknowledges awareness of the other while at the same time conveying the message that one is indifferent to her. In other words, the one claiming reserve must see, and therewith be reassured of, the other’s indifference. Without this acknowledgment of nonacknowledgment, civil inattention has not been achieved. It is this element that makes civil inattention stand out among the correlate notions of restraint. It is also, as we shall see shortly, the element that is particularly challenging for practices of social behaviour when consumer-based FR enters the picture.

## Facial recognition and the impairment of civil inattention

FR, the technology alluded to in our scenario, is a technology capable of identifying a person from a digital image by comparing a photographed face to a database of faces, and selecting the face with the best match. Although the technology is far from fool-proof, the accuracy of FR is improving, and it is not unrealistic to expect that FR apps on smartphones will soon technically be able to identify people on the tram with reasonable success rates (Acquisti et al. 2014; Welinder and Palmer 2018). Like many other networked technologies, FR has spurred heated debate and disagreement about its benefits and risks. Supporters anticipate that it will make the world a safer place by helping track down criminals and terrorists, detect shoplifters, and find missing children, that it will improve education and quality of care, and that it will render shopping experiences more convenient (Bonilla et al. 2019; Carter 2018; Interpol 2020; Oxagile 2017). Critics apprehend numerous and far-reaching risks, including facilitation of harassment and violence by law enforcement, the curtailing of civil liberties and fundamental rights and a general chilling effect on human freedom and flourishing, all with disproportionate negative impacts on people of colour and other minorities (Browne 2015; Garvie et al. 2018; Lynch 2018). Critics have thus likened FR to “nuclear waste” (Stark 2019) and described it as “the most uniquely dangerous surveillance mechanism ever invented” (Hartzog and Selinger 2018), with many calling for tight regulation or an outright ban on the technology.^4^

### Consumer use of FR as a challenge to behavioural-informational privacy

Daniel Solove (2008) suggests that, rather than pondering the nature of privacy in the abstract, we should try to understand concrete problems that create a desire for privacy, and only then draw on theory to better elucidate and address such problems. What kind of privacy problem does the use of a technology like FR constitute, particularly in the context of interaction between individuals such as the one depicted in our tram scenario? Which type of privacy in other words, does a technology like consumer-based FR challenge?

**Fig. 3:**
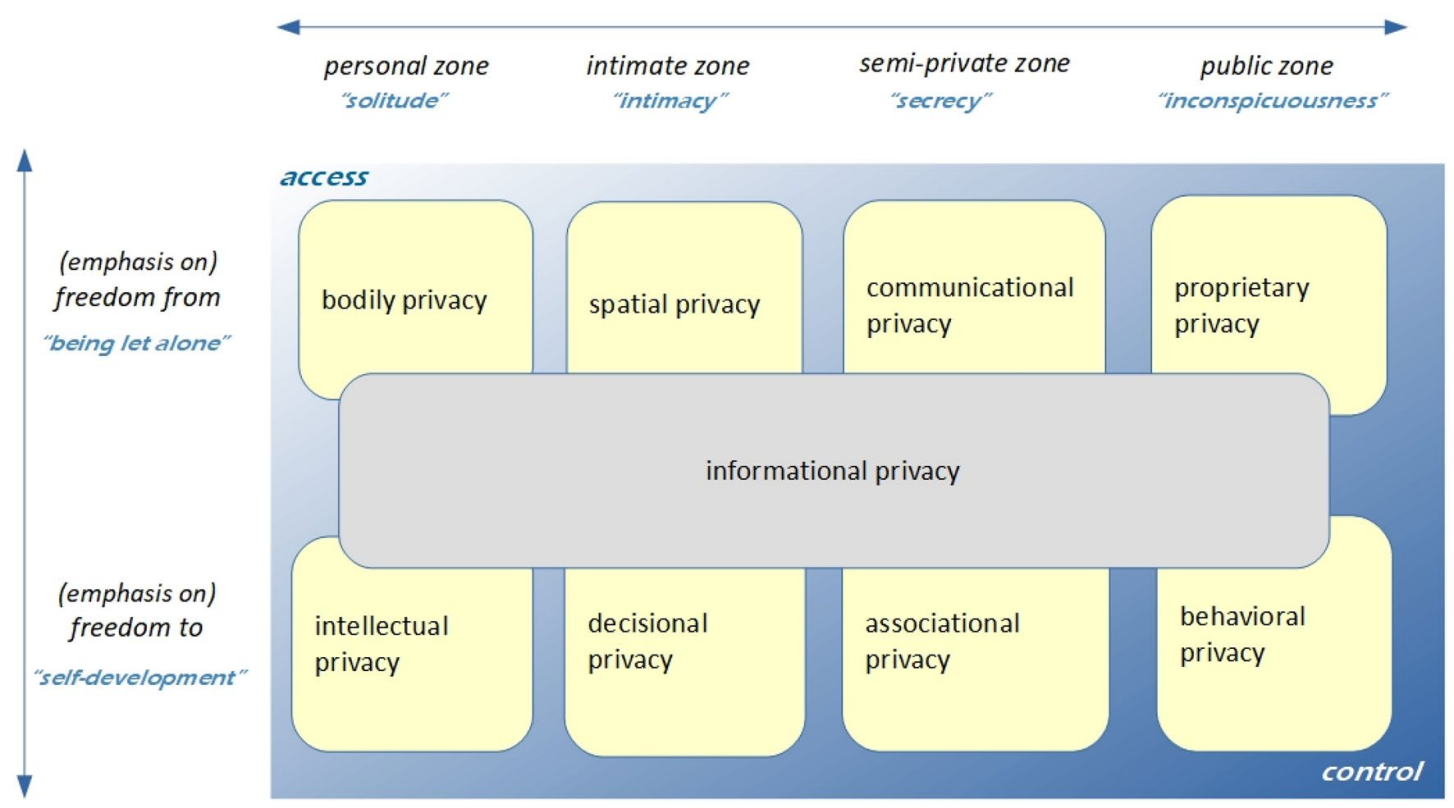
Typology of privacy. (*Source* Koops et al. 2017, p. 566)

Several authors have proposed taxonomies or typologies to capture the complex, multifaceted nature of privacy and to distinguish types of privacy (e.g. Clarke 1997/2016; Finn et al. 2013). The most comprehensive framework to date, integrating earlier classifications, is the privacy typology developed by Koops et al. (2017) (see Fig. 3).

Koops et al.’s typology includes eight basic types of privacy: bodily, spatial, communicational, proprietary, intellectual, decisional, associational and behavioural privacy. Over these eight basic privacy types is an overlay of informational privacy, as the authors argue that all types of privacy have an informational component, but that they cannot be solely reduced to this component. These privacy types are positioned along two main axes: an axis of social interaction (ranging from the strictly personal and intimate to the semi-private and public), and an axis of freedom (ranging from the notion of negative freedom associated with being let alone to the notion of positive freedom of self-development).

Along Koops et al.’s axis of social interaction, the main challenge consumer-based FR technology poses to privacy lies towards the public side of the spectrum. Occasionally, FR might raise awkward questions in the intimate sphere, for example when a mother checks the Internet profile of every classmate her teenage daughter brings home. Consumer-based FR will more often trigger issues in semi-private contexts where people traditionally benefit from anonymity (e.g., in Alcoholics Anonymous meetings, the audience of a large lecture hall). But overall, consumer-based FR will have the largest impact in publicly accessible spaces, particularly in urban areas where people traditionally move around with an expectation of inconspicuousness (the primary characteristic that Koops et al. associate with the public zone).

Along the axis of freedom, positive freedom—the freedom to act autonomously in public (within boundaries of social and legal acceptability)—is what is most at stake in the use of consumer-based FR in publicly accessible spaces. This is not to say that negative freedom will not be affected by FR: the capacity of people to ward off interference by others (e.g. by drawing up some shield around them), may require new and complicated forms of boundary management in public spaces. Generally, however, the capacity for boundary management in publicly accessible spaces is limited, and the basic privacy type Koops et al. articulate at the intersection of negative freedom and the public zone—that of proprietary privacy (e.g., using a handbag to shield personal items from public interference)—does not seem well-suited to deal with consumer-based FR. It is thus at the intersection of the public zone of life and the positive freedom of self-development that the privacy problem raised by consumer-based FR should be situated. In other words, it is a combination of *behavioural privacy*, as the ideal-typical privacy interest a person has while conducting publicly visible activities, and *informational privacy*, typified by the interest in preventing information about oneself to be collected and in controlling information about oneself that others may have legitimate access to, which are the key types of privacy challenged by consumer-based FR. It is precisely this type of behavioural-informational privacy that civil inattention and the correlated norms of reserve, discretion and nonacknowledgment seek to preserve.

### FR-enabled evasions of the rules of civil inattention

Goffman writes, “civil inattention is so delicate an adjustment that we may expect constant evasion of the rules regarding it” (1963, p. 85). As mentioned, civil inattention is performed mainly in the visual register. Artefacts that upset the mutual visibility required for civil inattention—that allow one to “steal glances” without being seen—enable such evasions. Writing before the digital era, Goffman speaks for example of dark sunglasses, fans and parasols. Consumer-based FR can further upset mutual visibility in several ways.

First, smartphone cameras allow for “stealing glances” in new ways. By acting as an additional eye, which can move around with more degrees of freedom than the eyes in one’s head, they increase *visibility*. Secondly, and more importantly, FR allows for more knowability and *recognisability*, which goes significantly beyond the surveillance that became possible when people began carrying around mobile cameras. The presence of mobile cameras opens up possibilities for snapshots—stolen glances that are fixed in pictures. However, those pictures are one-off images of one’s present behaviour; they may be awkward and give a feeling of discomfort, but they only have a bearing on one’s current behaviour and self-presentation. FR, in contrast, is the key that opens up many of one’s past stages of life to strangers, so that one has far less control over the impressions one gives off. In other words, if civil inattention is a courtesy that is granted and communicated in the gaze—specifically by *not* exercising the full functionality of the eye—FR expands this functionality beyond eyesight to include entire databases of personal information, unlocking whole new stages of past performances, and far surpassing the capacities of human memory.

Thirdly, consumer-based FR evades the rules of civil inattention insofar as it is not clear how showing or expressing indifference can be done with a smartphone. One crucial difference between eye-based civil inattention—the fleeting recognition of the other that expresses disinterest in the other—and a FR app is that the camera’s eye may be pointed equally fleetingly, but that disinterest can never be expressed. When another person moves her eyes away after glancing at you for a split second, you know that you are no longer observed. But the photograph that may have been made in the split second, in contrast, may be the starting point of further in-depth scrutiny of your person. Or not. There simply is no way of knowing. While we still may have a reasonable expectation that others in our vicinity in publicly accessible spaces are not overly (nor unduly) interested in us, there is no way of knowing what the others’ smartphone cameras are registering. It is this combination of FR’s potential to open up to strangers our past performances that are present online, with the lack of strangers’ ability to *show* that this is not what they are actually interested in, that makes consumer-based FR so evasive of civil inattention and disruptive of behavioural-informational privacy.

## Shortcomings in academic and regulatory approaches with regards to the privacy problem of consumer-based FR

In this section we argue that the focus on behavioural-informational privacy and civil inattention as a means of protecting it highlights some limitations within prevalent academic and regulatory approaches for addressing the privacy problem of consumer-based FR. This foregrounds the importance of revitalising the concept of civil inattention in these discussions, of paying increased attention to the role of social norms in privacy preservation and to the careful distribution of responsibilities in the aim of accomplishing privacy in public.

### Informational models

Dominant theoretical approaches to privacy today tend to be informational models of privacy, where privacy pertains to the control over, management or restricted access to, private information (Fried 1968; Nissenbaum 2010; Solove 2015; Westin 1967). Insofar as the privacy problem raised by consumer-based FR is in part informational, informational models of privacy are helpful for grasping it. In a very straightforward way, and in terms of the privacy dyad presented in Fig. 1 above, use of an FR app grants person *A* access to personal information on person *B* without her explicit consent. Consumer-based FR software finds its place here alongside other increasingly ubiquitous automated systems, including CCTV and WiFi tracking, that enable the collection of information about people in public spaces. As Gary Marx (2015, p. 41) writes, the generic activity of surveillance is often understood as “the taking-in of data”, and in its most recent form, the use of technology to “extract or create information” on individuals and groups in ways that “go beyond what is naturally offered to the senses and minds unsupported by technology”. In this way, consumer-based FR can be understood as a technical means of enhancing naturally occurring forms of data extraction like seeing, hearing or smelling, and of enabling privacy infringements understood as the loss of the capacity of individuals to exercise control over their personal information in terms of what they display to others when moving around in public space. But informational models of privacy are insufficient for grasping in full the privacy problem raised by consumer-based FR.

This is because the privacy problem engendered by consumer-based FR exceeds informational privacy. Privacy extraction, or even information flowing inappropriately between contexts (Nissenbaum 2010), is not all that is at stake. In our tram scenario, if the FR app being used to identify your face were to come up with an incorrect match, or if some malfunction prevented it from connecting to the cloud, in which case no extraction of personal information would actually take place, it would still constitute an interference with your privacy. Feelings of anxiety and discomfort are to be expected in such a situation even if no personal information is made accessible.

### Panoptic surveillance

Here, surveillance theories may be more helpful: the panoptic effects of surveillance and its power as a technology of social control emanate precisely from the fact that the watch tower may as well be empty (Foucault 1995). In this case, no actual flows of information are taking place. Yet the feeling of being watched, while not entirely unrelated to the taking-in of data, can produce far-reaching effects that do not only pertain to informational harms, such as blacklisting, identity theft or algorithmic discrimination. It can also have negative impacts on freedom, creativity and self-development (Cohen 2000; Gavison 1980; Schwartz 1999). Indeed, finding oneself to be the object of scrutiny of another’s attention has a chilling effect on freedom, individuality and self-determination. This relationship between privacy and autonomy that comes to the fore in surveillance theories brings us somewhat closer to what seems to be at stake in the behavioural-informational privacy problem posed by consumer-based FR.

At the same time, the privacy problem posed by FR, when it is used in settings of interactions between individuals such as our tram scenario, does not entirely fit within a panoptic surveillance framework either. A core dimension of the surveillance framing is the experience of an already existing inherently asymmetrical power relation between watchers and watched. From prisons and hospital wards to national security agencies and corporate actors, common surveillance settings usually entail an a priori power asymmetry, which is enhanced by, but typically *precedes* the use of any technical means of surveillance. The use of FR technologies in settings like the tram scenario, however, while they may *introduce* a power differential, do not necessarily enhance existing ones, although this may be the case.^5^ This is not to say that people on the tram are all equals, but that they are not unequal in the way that governments as opposed to citizens, and tech corporations as opposed to consumers are, even if the use of FR in such a setting certainly initiates a new power differential between them.

### Privacy as immunity from the judgment of others

Jeffrey Johnson’s (1989) conceptualization of privacy in the form of immunity from the judgment of others may be more helpful than informational and surveillance theories of privacy for capturing the specific behavioural-informational privacy challenge of consumer-based FR. Johnson discusses the example of a peeping Sarah observing him naked through his bathroom window. Sarah also happens to be his physician (i.e. she has already seen him undressed), and she is a frequent house guest (i.e. she knows what his home looks like). In this event, there is no acquisition of new personal information, but neither is the sense of unease that is felt by Johnson the result of an established power asymmetry which would impinge in a clear-cut way on Johnson’s life chances. Privacy here has to do with the sense of being free from the judgment of others in matters that can involve shame and embarrassment.

Similarly to those theorists who draw a link between privacy and autonomy or self-determination, Johnson emphasizes that an awareness that one is being watched can alter how one behaves, as the result of a heightened sense of self-consciousness, of seeing oneself through another’s eyes. However, for Johnson, this privacy infringement has to do with the importance of being free from the judgment of others in matters that can involve (culturally-specific) shame and embarrassment, such as nudity, sexuality and excretion. It is the possibility of being *judged* that has inhibitory effects. This emphasis on moral judgment seems ill-fitted to the consumer-based FR privacy problem depicted in the tram scenario. But it does help explain the feeling of unease you may have with the woman on the tram identifying you. What will she think of you? Will she see you in a certain light, now that her view of you is not constituted solely by the pressed shirt you are wearing but also by the pictures of last week’s beach party? It is relevant to recall here that Goffman’s classic articulation of impression management—a key part of self-development in social life—focuses not so much on the impressions people *give* (e.g., in the information they expressly communicate to others), but rather on the impressions people *give off*, which are more of “the non-verbal, presumably unintentional kind” (Goffman 1959, p. 4).

### Legal instruments for privacy protection

**Fig. 4:**
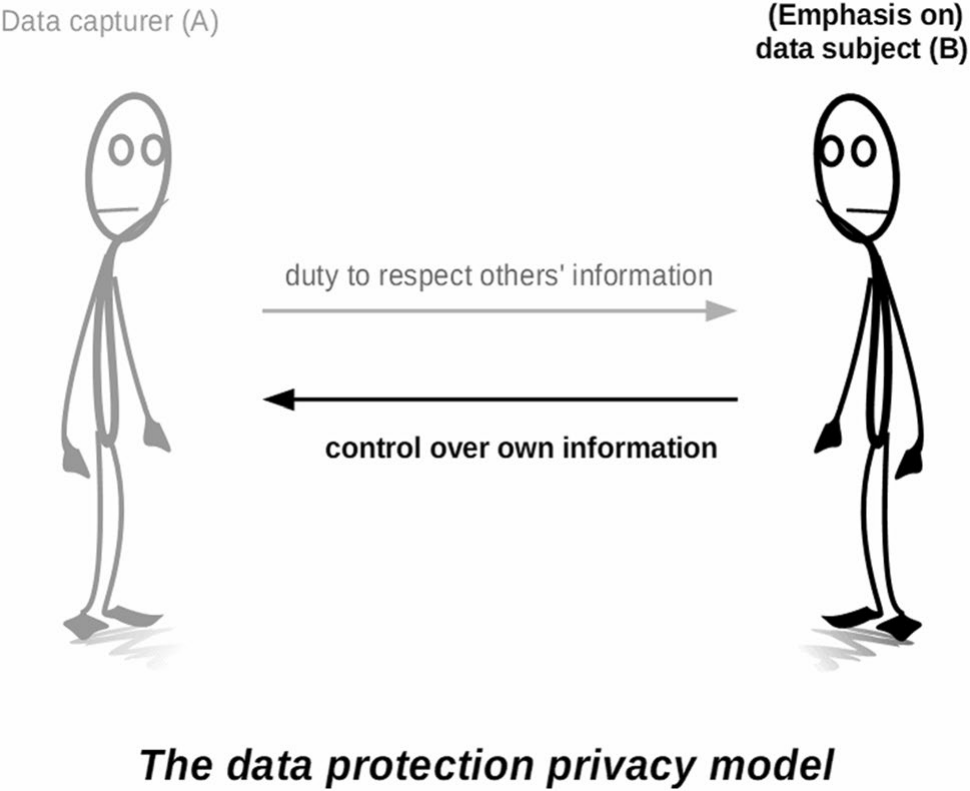
Distribution of responsibilities in the data protection privacy model

Mirroring the privileged place accorded to informational privacy in the theoretical scholarship, the primary regulatory lens applied to privacy problems in most if not all Western jurisdictions is, likewise, informational privacy. In addition to the theoretical problems consumer-based use of FR poses to informational models of privacy, as discussed above, current legal instruments present an additional shortcoming which the discussion on civil inattention brings to the fore: they do not do enough to account for the relational nature of privacy. Legal instruments such as the EU General Data Protection Regulation, but also attempts to apply traditional “notice and consent” frameworks to surveillance technologies (e.g. the FTC’s guidance on FR in the United States (2012, p. iii)), are aimed towards increasing and safeguarding the empowerment of data subjects. In terms of our privacy dyad, the emphasis is on person *B*, and protecting her by providing her more possibilities for controlling and deciding what can be done with her data (Fig. 4). This poses two problems with relation to civil inattention.

First, in the civil inattention model of privacy-protection, data subject empowerment translates into possibilities for achieving disattendability, which is indeed an important counterpart to a valid claim to civil inattention (cf. Fig. 2). But, as discussed, in a civil inattention model of privacy, privacy is achieved as a result of reciprocal and collaborative relations that involve efforts on the part of both parties involved. Disattendability alone is never enough to ensure privacy here, and it is furthermore questionable if it can achieve anything in the face of powerful technology such as FR. To some extent, people can shield things that would otherwise attract undesirable attention when moving around in public. For example, a blackeye may be hidden behind sunglasses, or chemotherapy-induced hair loss can be hidden with a wig or a scarf. In contrast, it is difficult if not impossible to draw up a shield against FR. Effective shields could take the form of either covering up most of one’s face (e.g. with a baclava, niqāb or medical face mask),^6^ or using obfuscating make-up that could thwart FR algorithms. Both these types of shields are (still) unusual in public spaces: balaclavas and niqābs are, in most Western societies, contested in terms of social (and sometimes legal) acceptance (Daly 2017), the hope is that the need for face masks will eventually dissipate, and Harvey’s (2017) CV Dazzle pictures showing FR-resistant camouflage show beautiful but distinctly weird forms of presenting one’s face to others. Anti-FR shields may serve as a useful purpose in demonstrating resistance to body-focused surveillance (Nagenborg 2017), but they do not scale up; such shields are likely to continue to attract more rather than less attention from others, thwarting the aim of achieving inconspicuousness. It is thus unrealistic (and morally questionable) to expect such shields to become common means of protecting oneself from FR technology.

Second, beyond shields, an important means of empowering data subjects within legal frameworks such as the GDPR is to grant data subjects the power to decide how others process information about her. But this theoretical ambition is far removed from practice, since in today’s information society people hardly have an overview of all the information flows, and little meaningful control in practice over all the information stored in the world’s vast databases (Koops 2014). This is particularly apparent in the data capture taking place in public, where people may attempt to manage the impressions they give off to others (e.g. by their clothes, gestures and movement), but can hardly control how others process the information about them derived from CCTV, WiFi tracking, and other sensors in public spaces. Models based on transparency—giving notice to the public if and how sensors such as cameras are being used—may provide some initial privacy protection, since people can decide to shun areas where undesirable data capture takes place, but will soon run into problems of scale. You simply cannot consistently avoid CCTV cameras. And while information control is already a challenge vis-à-vis government and corporate data capturers in public spaces, it is considerably more difficult vis-à-vis other individuals walking around in public space.

**Fig. 5:**
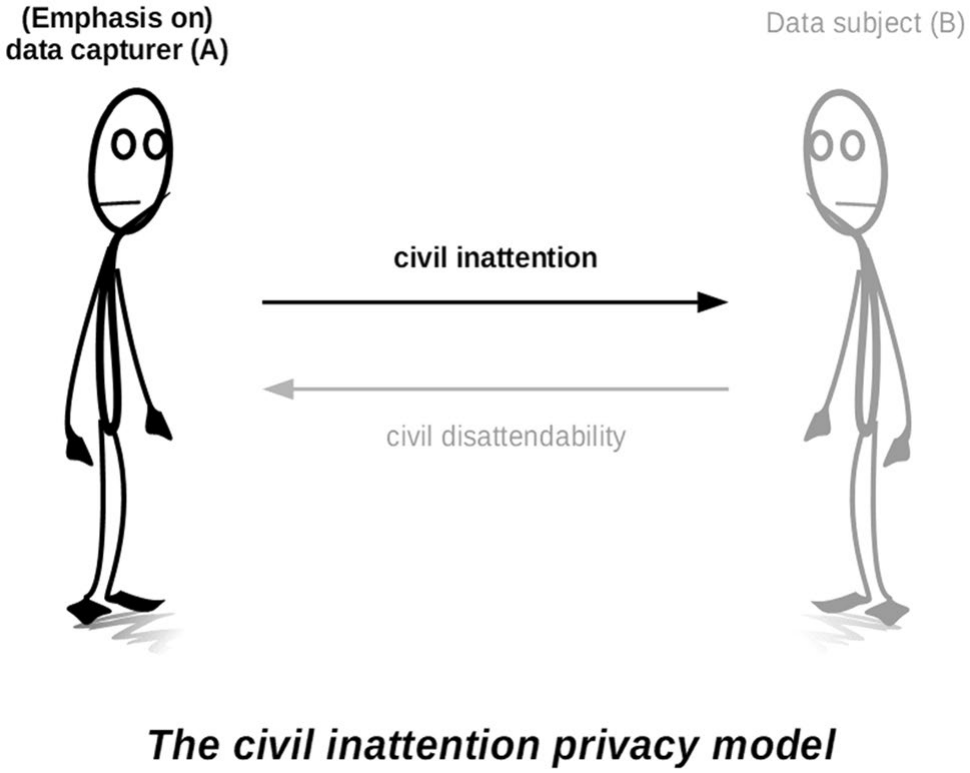
Distribution of responsibilities in the civil inattention privacy model

In other words, dominant legal frameworks that seek to empower data subjects are arguably insufficient in today’s information society because they focus too much on what data *subjects* can do, and too little on limiting what data *capturers* can (and, what is more, should) do. In terms of our privacy dyad, there is too little focus on the relational nature of privacy, and too much focus on person *B* to the detriment of person *A*.^7^ In contrast, a civil inattention model to privacy foregrounds the relational nature of privacy in public spaces and within this relationship places more responsibility on person *A*. And while it entails that both data capturers and data subjects engage in privacy-protecting relationships in order to accomplish behavioural-informational privacy, the onus—extending civil inattention—is placed on person *A* (Fig. 5). To be sure, this shortcoming of dominant legal frameworks is due in no small part to the difficulty of actually limiting what *A* can do in the light of ever more technological possibilities. But that is precisely why we believe norms such as civil inattention, as social forces that temper what is functionally possible, should deserve more attention in this predicament.

## Towards a civil inattention approach to privacy in the digital age

The argument we developed in this paper started with the observation that FR technology, especially its use by citizens or consumers, challenges privacy expectations in relation to practices of social behaviour, as illustrated in the example of someone on the tram who may (or may not) have just taken a picture of you, and may (or may not) now be browsing through whatever information is available about you online. In particular, we argued, FR impedes the ability to practice civil inattention and correlated norms, including discretion, nonacknowledgment, reserve and disattendability, which contribute to preserving behavioural-informational privacy. We stressed the particular characteristics of civil inattention, which expresses not only that indifference in public functions as a social norm (it is *civil* inattention) but also that the indifference needs to be shown (it is civil *in-attention*) in order to function as a privacy-preserving mechanism. It is the demonstration of indifference that is needed to reassure people in publicly accessible spaces that they have no reason to fear the judgment of others, and therewith can relax and behave as they wish (up to the point of impropriety that calls for attention). The indifference is shown and inferred through subtle clues, in particular eye and body movement that suggest awareness of, but not particular interest in, another’s person. By enacting civil inattention, we respect others as persons and we respect the room they need for behavioural-informational privacy. Yet, by increasing the visibility and knowability of those who watch others through their smartphone cameras, consumer-based FR upsets the mutual visibility and social reciprocity that are key to civil inattention. It disrupts the routine practices and moral act of civil inattention. Furthermore, we suggested that a focus on how the practice of civil inattention is disrupted by FR foregrounds two elements that deserve more attention in the academic and regulatory approaches to privacy and data protection, namely its relational nature and the extent to which it relies on social norms. Indeed, civil inattention entails a relational and collaborative effort, a delicate balance whereby one needs to demonstrate reserve or disattendability in order to receive nonacknowledgement or civil inattention from others in return, but where the onus is chiefly on data capturers to extend civil inattention, rather than on data subjects to practice civil disattendability.

### Limitations

Our analysis presents some inherent limitations. First, it does not account for diverse cultural and social factors that influence expectations of behavioural-informational privacy and civil inattention among individuals. These will diverge in different cultures (e.g. Mediterranean vs. Nordic societies) and situations (e.g. daytime vs. night time, emergencies, etc.). Second, power is all but absent in Goffman’s analysis of the interpersonal norms that govern social behaviour in public. Or rather, the practice of civil inattention already delimits a space which excludes normalized inequalities and injustices: the avoidance of beggars and psychiatric patients on the streets, or the Southern “hate stares” that Goffman mentions, are not the object of his scrutiny, nor is the question of who decides who is a potential candidate for deserved civil inattention. In order to develop a full-blown civil inattention approach to privacy in the age of digital surveillance, the question of power in its many dimensions would need to be addressed.

For example, as we discussed, civil inattention implies some assumed a priori level of equality between strangers. While this assumption of equality—or the assumption of the absence of an a priori inequality—between individuals is useful to point out where a civil inattention-informed approach to privacy diverges from panoptic surveillance approaches, the usefulness of this oversimplification is limited to this. There will certainly be individual inequalities around the use of consumer-based FT, including vectors such as age, gender and the financial ability to possess and upgrade smartphones. Furthermore, the additional actors who are always present beyond our dyadic models, namely governments and corporations, cannot remain absent from a complete analysis, which would need to investigate which cross-cutting and overlapping interests between these actors are also at work. Finally, important to note for any discussion on the ethics of inattention is the damage that it may do. As feminist critics of privacy have long argued (e.g. MacKinnon 1989), to the extent that the private sphere is held unavailable for public scrutiny, privacy protection can shield relations of domination and abuse towards women and other vulnerable groups who have traditionally been restricted to the public or domestic sphere.^8^

### The power of norms

Despite these limitations, we do feel that our analysis demonstrates the value of reviving the notion of civil inattention and associated norms as important contributions to the current academic and regulatory debate on privacy and surveillance technologies. Our portrayal of how civil inattention becomes increasingly difficult to enact in data-rich environments strengthens recent calls to strictly regulate or even ban FR technology, contributing to these an additional and heretofore neglected dimension of the privacy problem raised by FR. Furthermore, our analysis may help to point out some directions, pending strict regulation or outright bans, in which coping strategies can be sought. Indeed, the crucial role of social norms and practices in preserving privacy in public suggests that prohibitive laws or other command-and-control based forms of regulation are likely insufficient to prevent the impact of disruptive technologies on social relations in public space, and that a significant means of addressing this disruption will have to come from social practices. When it comes to technological disruption and the destabilization of routine moral practices, a recalibration of values and practices needs to take place (Swierstra 2013). This is not to say that laws are not called for, but that an additional and no less important way of dealing with the privacy problem raised by consumer-based FR, if it becomes pervasive, will be for society to develop new ways of enacting behavioural-informational privacy: to cultivate new ways of practicing civil inattention. Of course, the assumption here is that many people will find the need to preserve privacy in public important enough to adjust their behavioural practices and engage in the delicate and demanding choreography of civil inattention.

We can imagine, for example, that a norm might slowly evolve to not hold smartphones in such a way that bystanders might feel themselves in shooting range of the camera, and that where such positioning of the smartphone cannot be avoided, that some traditional fleeting eye contact is made to acknowledge awareness of and express indifference. Such behaviour could be assisted, perhaps, by design-based cues, such as a “civil inattention slider” that visibly shields off one’s phone camera when not in use, or a tiny flashing light that indicates the camera is in use in combination with a FR app.^9^ We do not know whether such new behavioural norms and design-based cues are realistic and effective, and in any case, social norms are slow to develop. Sometimes public-policy interventions may help the development or readjustment of social norms. Through agenda-setting and awareness-raising, policy-makers could seek to make both users and FR developers aware of the disruptive consequences of consumer-based FR, and therewith stimulate the development of new practices of smartphone-camera-sensitive civil inattention.

Such an adjustment of social practices and the evolving of new social norms is challenging, but not unprecedented. In his study of how the introduction of railway travel altered our perceptual experience, Wolfgang Schivelbusch (1986) described the development of a “panoramic gaze”, whereby people travelling on trains learned to gaze far away in order to take in a rapidly moving landscape. Similarly, Stefan Hirschauer (2005), drawing on Schivelbusch in his study of how civil inattention is practiced in elevators, describes the development of a short-sighted “cage gaze” in elevator travel, whereby people in elevators vacantly gaze, neither seeing nor communicating.

It is a long shot, but just possibly, something like a “smartphone gaze” might emerge in a future consumer-based FR-pervasive world, which would enable people moving around in public to spot smartphones in their surroundings that have them in shooting range, and then display—through bodily clues or props—a state of reserve that indicates a desire not to be the object of FR. If practices of civil inattention would co-evolve with such a smartphone gaze and FR-sensitive expressions of reserve, smartphone users might be able to exercise a proper level of discretion by not only refraining from using FR against bystanders, but also by somehow being able to demonstrate their indifference. Perhaps, if the woman on the tram had sought some brief eye contact with you and then displayed her disinterest in you by staring outside rather than at her smartphone screen, you would already have felt a little less uncomfortable when the possibility that she was face-recognising you flashed through your mind.

## Footnotes

1 In 1959, Goffman already wrote of “tactful inattention”: “when interaction must proceed in the presence of outsiders, outsiders tactfully act in an uninterested, uninvolved, unperceiving fashion, so that if physical isolation is not obtained by walls or distance, effective isolation can at least be obtained by convention” (1959, p. 230).

2 We are aware that civil inattention is a norm characteristic of Western urban forms of social interaction, which may not be as present in other non-Western societies, and that the increased cultural diversity of Western urban societies will influence practices and expectations of civil inattention. Accounting for these influences is beyond the scope of the analysis here.

3 Westin’s other states of privacy are solitude, intimacy, and anonymity.

4 Currently, FR regulation varies globally. In 2018 the city of San Francisco banned the use of FR by police forces, and at the time of writing the European Commission was considering a temporary ban on FR use in public areas (Spadafora 2020). Especially in the wake of the “Black Lives Matter” protests in the summer of 2020, there has been increasing pressure to ban the use of FR technology by law enforcement, and a number of large tech companies declared they would stop producing general-purpose FR and analysis software (IBM), refuse to sell FR technology to police agencies (Microsoft), or impose a moratorium on the development of the technology (Amazon) (Heilweil 2020).

5 While this distinction between state, commercial and consumer types of surveillance is to some extent artificial, and while the use of consumer-based FR may certainly reinforce existing inequalities along age, gender, cultural and socio-economic lines, this distinction remains useful for highlighting where a civil inattention approach to privacy may differ from a panoptic surveillance one.

6 At the time of writing, reports were emerging that the use of face masks for containing the spread of SARS-CoV-2 reduced the accuracy of FR (Vincent 2020).

7 Whether this focus on person *B* is an inherent characteristic of informational privacy, or the effect of the individual rights-based discourse in which discussions on privacy predominantly take place today, is a discussion that lies beyond the scope of this paper.

8 Nonetheless, problematising the public/private distinction and condemning the abuses that privacy can shield does not entail that there should be *no* private realm (for women as for everyone else), and other feminist approaches have argued for preserving this distinction while freeing it from its gendered bias (see e.g. DeCew 2015). A civil inattention approach to privacy implies that there is value in the public/private distinction, also *within* publicly accessible spaces, but acknowledges that to be ethical, inattention must be *civil*, which entails that it does not sanction inattention towards violence, abuse and exploitation.

9 See for example the design-based solution developed in Japan to prevent upskirt photography, in the form of a mandatory camera shutter sound that bystanders can hear whenever a picture is made. Of course, such design-based solutions may be circumvented, as happened when people were offered tweaks to prevent the sound from sounding (e.g. https://www.unlockboot.com/turn-off-camera-sound-on-iphone-to-take-silent-photo/). But a design cue can serve at least as a form of privacy-by-default, and could also be a starting point for the evolution of new social norms (for instance, that it is considered inappropriate to circumvent the mandatory sound in certain social settings).

## References

[CR1] Acquisti A, Gross R, Stutzman F (2014). Face recognition and privacy in the age of augmented reality. Journal of Privacy and Confidentiality.

[CR2] Arendt H (1958). The human condition.

[CR3] Benjamin W (2002). The Arcades Project.

[CR4] Bonilla S, Moguel E, Garcia-Alonso J, García-Alonso J, Fonseca C (2019). Facial recognition of emotions with smartphones to improve elder quality of life. International Workshop on Gerontechnology.

[CR5] Browne S (2015). Dark matters: On the surveillance of blackness.

[CR6] Carter, A. M. (2018). *Facing reality: The benefits and challenges of facial recognition for the NYPD*. Retrieved February 6, 2020, from https://apps.dtic.mil/docs/citations/AD1065272.

[CR7] Clarke, R. (1997). *Introduction to dataveillance and information privacy, and definitions of terms*. Roger Clarke’s Home-Page. Retrieved February 6, 2020, from http://www.rogerclarke.com/DV/Intro.html.

[CR8] Cohen J (2000). Examined lives. Stanford Law Review.

[CR9] Daly A, Tjerk T, Newell BC, Koops BJ (2017). Covering up: American and European legal approaches to public facial anonymity after *SAS* v. *France*. Privacy in public space.

[CR10] DeCew JW, Roessler B, Mokrosinska D (2015). The feminist critique of privacy. Social dimensions of privacy.

[CR11] Dewey J (1954). The later works.

[CR13] Finn RL, Wright D, Friedewald M, Gutwirth S, Leenes R, de Hert P, Poullet Y (2013). Seven types of privacy. European data protection: Coming of age.

[CR14] Foucault M (1995). Discipline and punish.

[CR15] Fried C (1968). Privacy. The Yale Law Journal.

[CR16] FTC (Federal Trade Commission) (2012). Facing facts. Best practices for common uses of facial recognition technologies.

[CR17] Garvie, C., Bedoya, A., & Frankle, J. (2018). The perpetual line-up: Unregulated police face recognition in America. *Perpetual Line-Up*. Retrieved February 6, 2020, from https://www.perpetuallineup.org/.

[CR18] Gavison R (1980). Privacy and the limits of law. The Yale Law Journal.

[CR19] Goffman E (1959). The presentation of self in everyday life.

[CR20] Goffman E (1963). Behaviour in public places. Notes on the social organizations of gatherings.

[CR53] Gouinlock, J. (Ed.). (1994). *The moral writings of john dewey*. Amherst, NY: Prometheus.

[CR21] Hartzog, W., & Selinger, E. (2018). Facial recognition is the perfect tool for oppression. *Medium.* Retrieved February 6, 2020, from https://medium.com/s/story/facial-recognition-is-the-perfect-tool-for-oppression-bc2a08f0fe66.

[CR22] Harvey, A. (2017). *Camouflage from face detection*. Retrieved February 6, 2020, from https://cvdazzle.com/.

[CR23] Heilweil, R. (2020). Why it matters that IBM is getting out of the facial recognition business. *Vox*. Retrieved October 6, 2020, from https://www.vox.com/recode/2020/6/10/21285658/ibm-facial-recognition-technology-bias-business.

[CR24] Hirschauer S (2005). On doing being a stranger. Journal for the Theory of Social Behaviour.

[CR25] Interpol. (2020). *Facial recognition*. Retrieved February 6, 2020, from https://www.interpol.int/How-we-work/Forensics/Facial-Recognition.

[CR26] Johnson J (1989). Privacy and the judgement of others. The Journal of Value Inquiry.

[CR54] Keulartz J, Schermer M, Korthals M, Swierstra T (2004). Ethics in a technological culture: A pragmatist proposal for a pragmatist approach. Science, Technology & Human Values.

[CR27] Koops BJ (2014). The trouble with European data protection law. International Data Privacy Law.

[CR28] Koops BJ, Newell B, Timan T, korvánek I, Chokrevski T, Galič M (2017). A typology of privacy. University of Pennsylvania Journal of International Law.

[CR29] Kudina O, Bas M, Newell BC, Timan T, Koops BJ (2018). ‘The end of privacy as we know it’: Reconsidering public space in the age of Google Glass. Surveillance, privacy and public space.

[CR30] Lynch, J. (2018). *Face off: Law enforcement use of face recognition technology*. EFF. Retrieved February 6, 2020, from https://www.eff.org/wp/law-enforcement-use-face-recognition.

[CR31] MacKinnon C (1989). Toward a feminist theory of the state.

[CR55] Marx G (2015). Surveillance studies. International Encyclopedia of the Social & Behavioral Sciences.

[CR32] Nagenborg M, Tjerk T, Newell BC, Koops BJ (2017). Hidden in plain sight. Privacy in public space.

[CR56] Nagel T (1998). Concealment and exposure. Philosophy & Public Affairs.

[CR33] Nissenbaum H (2010). Privacy in context: Technology, policy, and the integrity of social life.

[CR34] Oxagile. (2017). *Is face recognition the key to a safe and responsible world?* Retrieved February 6, 2020, from https://www.oxagile.com/article/is-face-recognition-the-key-to-a-safe-and-responsible-world/.

[CR35] Roessler B, Mokrosinska D (2013). Privacy and social interaction. Philosophy and Social Criticism.

[CR36] Schivelbusch W (1986). The railway journey.

[CR37] Schwartz P (1999). Privacy and democracy in cyberspace. Vanderbilt Law Review.

[CR38] Sharon T (2017). Self-tracking for health and the quantified self. Philosophy & Technology.

[CR39] Simmel G (1906). The sociology of secrets and secret societies. American Journal of Sociology.

[CR40] Simmel G, Bridge G, Watson S (2002). The metropolis and mental life. The Blackwell city reader.

[CR41] Solove DJ (2008). Understanding privacy.

[CR42] Solove DJ, Roessler B, Mokrosinka D (2015). The meaning and value of privacy. Social dimensions of privacy.

[CR43] Spadafora, A. (2020). *EU calls for five year ban on facial recognition*. TechRadar. Retrieved February 6, 2020, from https://www.techradar.com/news/eu-calls-for-five-year-ban-on-facial-recognition.

[CR44] Stark, L. (2019). *Facial recognition is the plutonium of AI*. XRDS: Crossroads. Retrieved February 6, 2020, from https://xrds.acm.org/article.cfm?aid=3313129.

[CR45] Swierstra T (2013). Nanotechnology and technomoral change. Ethics & Politics.

[CR46] Tonkiss F (2003). The ethics of indifference. International Journal of Cultural Studies.

[CR47] Vincent, J. (2020). Face masks are breaking facial recognition algorithms, says new government study. *The Verge.* Retrieved October 6, 2020, from https://www.theverge.com/2020/7/28/21344751/facial-recognition-face-masks-accuracy-nist-study.

[CR49] Welinder Y, Palmer A, Selinger E, Polonetsky J, Tene O (2018). Face recognition, real-time identification, and beyond. The Cambridge handbook of consumer privacy.

[CR50] Westin AF (1967). Privacy and freedom.

[CR51] Wirth L (1938). Urbanism as a way of life. American Journal of Sociology.

